# Molecular Phenotypes Distinguish Patients with Relatively Stable from Progressive Idiopathic Pulmonary Fibrosis (IPF)

**DOI:** 10.1371/journal.pone.0005134

**Published:** 2009-04-06

**Authors:** Kathy Boon, Nathaniel W. Bailey, Jun Yang, Mark P. Steel, Steve Groshong, Dolly Kervitsky, Kevin K. Brown, Marvin I. Schwarz, David A. Schwartz

**Affiliations:** 1 National Institute of Environmental Health Sciences/National Heart Lung and Blood Institute, Research Triangle Park, North Carolina, United States of America; 2 Pulmonary, Allergy, Critical Care Medicine, Duke University Medical Center, Durham, North Carolina, United States of America; 3 National Jewish Health, Denver, Colorado, United States of America; 4 University of Colorado Health Sciences Center, Denver, Colorado, United States of America; Helmholtz Zentrum München /Ludwig-Maximilians-University Munich, Germany

## Abstract

**Background:**

Idiopathic pulmonary fibrosis (IPF) is a progressive, chronic interstitial lung disease that is unresponsive to current therapy and often leads to death. However, the rate of disease progression differs among patients. We hypothesized that comparing the gene expression profiles between patients with stable disease and those in which the disease progressed rapidly will lead to biomarker discovery and contribute to the understanding of disease pathogenesis.

**Methodology and Principal Findings:**

To begin to address this hypothesis, we applied Serial Analysis of Gene Expression (SAGE) to generate lung expression profiles from diagnostic surgical lung biopsies in 6 individuals with relatively stable (or slowly progressive) IPF and 6 individuals with progressive IPF (based on changes in DLCO and FVC over 12 months). Our results indicate that this comprehensive lung IPF SAGE transcriptome is distinct from normal lung tissue and other chronic lung diseases. To identify candidate markers of disease progression, we compared the IPF SAGE profiles in stable and progressive disease, and identified a set of 102 transcripts that were at least 5-fold up regulated and a set of 89 transcripts that were at least 5-fold down regulated in the progressive group (P-value≤0.05). The over expressed genes included surfactant protein A1, two members of the MAPK-EGR-1-HSP70 pathway that regulate cigarette-smoke induced inflammation, and Plunc (palate, lung and nasal epithelium associated), a gene not previously implicated in IPF. Interestingly, 26 of the up regulated genes are also increased in lung adenocarcinomas and have low or no expression in normal lung tissue. More importantly, we defined a SAGE molecular expression signature of 134 transcripts that sufficiently distinguished relatively stable from progressive IPF.

**Conclusions:**

These findings indicate that molecular signatures from lung parenchyma at the time of diagnosis could prove helpful in predicting the likelihood of disease progression or possibly understanding the biological activity of IPF.

## Introduction

Idiopathic Pulmonary Fibrosis (IPF) is a chronic progressive disease of unknown etiology that is characterized by irreversible scarring in the lung. IPF is one of a subgroup of the diffuse parenchymal lung diseases (DPLD) of unknown origin, represented by the idiopathic interstitial pneunomias (IIPs). IPF is the most common form of IIP, and pathologically is represented by usual interstitial pneumonia (UIP) [1]–[3]. While hypotheses have been put forth, varying from chronic inflammation leading to widespread fibrosis to abnormal wound healing and deregulated epithelial cell function [4]–[9], the basic mechanism of disease pathogenesis remains unknown.

Disease progression is highly variable in IPF. While the 3 to 5 year mortality is 50%, this is quite variable with some patients living up to 10 years following diagnosis [10]. The disease course is also variable, ranging from patients who remain stable for protracted periods of time to others whom experience rapid stepwise progression with accelerated mortality [11]–[13]. Although predictors of survival [10] and disease progression [14] have included demographic factors, exposures, lung physiology, radiography, and pathology, it remains difficult to predict the prognosis of any one case of IPF. Moreover, none of the prediction models have accounted for differences in molecular features of the pathological process.

Unfortunately, patients generally present in the later stages of disease. And no medical treatment either reverses or slows the progression of IPF. This heterogeneity of disease progression and the lack of available treatment emphasize the importance of early diagnosis, especially with the hope that intervention may be more effective in the early stages of disease. This also underscores the need for biomarkers which not only may predict progression but may contribute to discovery of molecular mechanisms that are involved in disease pathogenesis.

We hypothesized that by comparing the transcriptome of relatively stable and progressive IPF, markers of disease activity would be identified that could lead to biomarker discovery, improved prognostic ability, and further contribute to the understanding of IPF pathogenesis. In this study, we generated the lung expression profiles from pre-treatment, diagnostic surgical lung biopsies using SAGE technology [15] from 6 individuals with relatively stable (or slowly progressive) IPF and compared these profiles to 6 individuals with progressive IPF. *In silico* analyses of the comprehensive SAGE profiles allowed for the generation of an IPF molecular signature that distinguished relatively stable from progressive patients, and identified genes not previously implicated in IPF. Moreover, the SAGE IPF gene expression profile identified molecular pathways that may be important in disease development and progression.

## Results

**Figure 1:**
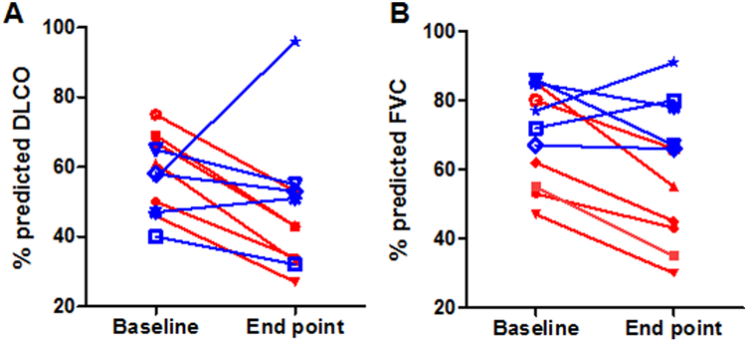
Forced vital capacity (FVC) and carbon monoxide diffusing capacity (DLCO) values. The percent predicted DLCO (A) and FVC (B) values are indicated at baseline and end point for the two IPF disease groups. The progressive group is represented in red and the relatively stable group in blue.

A summary of the clinical and demographic features are presented in **Table 1**. The average age was 64.8 years in the progressive group and 66.7 years in the relatively stable group. Both groups included smokers and non-smokers. However, only one female subject was present in the progressive group, whereas 3 were included in the relatively stable group (**Table 1**). The percent predicted pulmonary function test (PFT) values at baseline and end point for both groups are depicted in **Figure 1**. The mean of the percent predicted PFT values at baseline are not significantly different between both groups (**Table 1**). The actual PFT values are depicted in **Figure S1**. A significant difference between the progressive and the relatively stable group was found for the actual change in DLCO and the change in percent predicted DLCO with a P-value<0.05 based on a Mann-Whitney test. Given that not all samples were collected at equal time intervals between baseline and end point, a time-weighted factor was calculated to assure the correct group assignment. The time-weighted change in % predicted values between the two groups was significantly different with P-values between 2.2E-3 (DLCO) and 8.7E-3 (FVC).

**Table 1:** Clinical and demographic variables

Variable	Progressive group (n = 6)	Relatively Stable group (n = 6)
Age	64.8±6.7	66.7±5.5
Sex male/female	5/1	3/3
**Smoking status**		
never	3	2
ever	3	4
current	1	1
% predicted DLCO baseline	58.7±11.0	56.0±10.9
Actual change in DLCO	−6.4±1.5	−2.50±3.34
Change in % predicted DLCO	−20.8±4.8	−2.33±21.77
% predicted FVC baseline	61.2±18.1	68.0±16.9
Actual change in FVC	−0.73±0.4	−0.24±0.5
Change in % predicted FVC	−16.0±7.8	−4.17±13.9

The percent predicted DLCO and FVC values at baseline, as well as the changes in actual and predicted (DLCO or FVC) values over a 12 months period are indicated as the mean with standard deviation for each group.

### IPF SAGE Transcriptome

**Figure 2:**
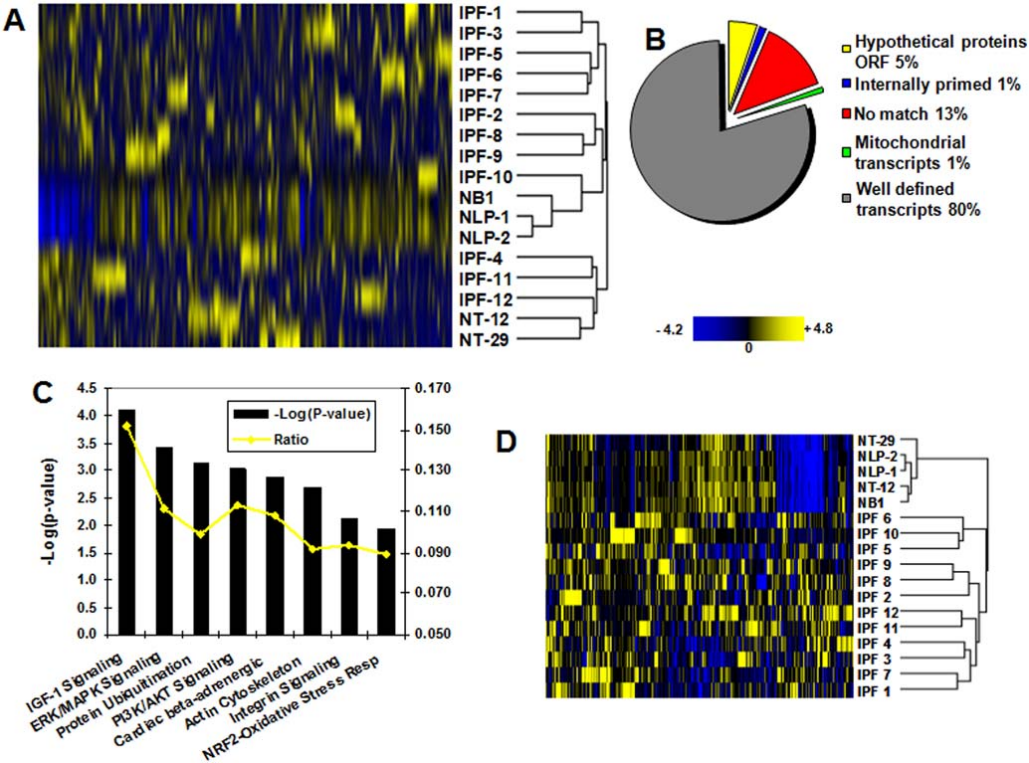
Analysis of the IPF transcriptome. (A) Hierarchical clustering based on the gene expression profiles of 12 IPF and 5 normal lung SAGE libraries described in Table S1. The branch length in the dendrogram represents the distance or relatedness between the samples; the shorter the branch the higher the similarity between samples. In yellow are indicated up regulated and in blue down regulated genes. (B) Tag to gene mapping classification of the 1,121 transcript tags significantly over expressed in IPF when compared to normal lung parenchyma. (C) Most significant canonical pathways associated with pulmonary fibrosis according to the IPA pathway analysis tool. The significance of the association between the dataset and the canonical pathway was measured as a ratio (number of genes from the dataset that map to the pathway divided by the total number of molecules that exist in the canonical pathway). A Fischer's exact test was used to calculate a P-value. (D) Hierarchical clustering based on the 293 transcriptional signature that distinguished IPF from normal lung parenchyma.

For an in-depth assessment of the IPF transcriptome, we generated and analyzed 12 IPF SAGE libraries with an average of 79,578 tags per library. A total of 954,932 transcript tags were sequenced of which 168,272 were unique. After removal of linker and repetitive sequences the number was reduced to 168,066. Transcript tags with a raw count of one in the entire IPF transcriptome (singletons) were also removed resulting in 149,291 transcripts. For comparison purposes, the tag counts in each library were normalized to 200,000 tags. We also included 8 other human lung tissue libraries comprising another 500,244 tags that were downloaded from the SAGE Genie (http://cgap.nci.nih.gov/SAGE) or the GEO website (http://ncbi/geo/). All libraries included in this study are described in **Table S1**. Hierarchical clustering analysis of the 12 IPF and 5 normal lung parenchyma SAGE libraries included in this study demonstrated that IPF samples are distinguishable from normal lung parenchyma (**Figure 2A**). Three IPF samples are clustered together and are dissimilar from the other 9 IPF samples indicating the possible existence of other subtypes and reflecting the heterogeneity among IPF patients. Though the three samples belong to the relatively stable group (**Table S1**), it cannot be excluded that this separation might be simply due to normal lung parenchyma present within the surgically removed biopsy sample. We repeated the unsupervised clustering with all 22 SAGE libraries available and noticed that these particular three samples are still closer related to certain normal tissue samples though the separation between IPF and normal lung parenchyma is not as efficient as in the first analysis (**Figure S2A**).

Initially, we compared the normal lung parenchyma libraries NB1, NLP-1 and NLP-2 with the 12 IPF SAGE libraries in order to identify genes that are over expressed in IPF and are minimally expressed or absent in normal lung parenchyma. The filter applied for each individual transcript tag selected for a P-value≤0.05 and a fold difference of at least 10; less than 2 counts in the normal libraries; 10 or more counts in the IPF group; and an expression level of at least 5 counts in 50% of all the IPF SAGE libraries, which resulted in 1,121 transcript tags. The tag to gene mapping was performed using SAGE Genie downloads and the tag to gene classifications of the 1,121 transcript tags shows that 80% of the tags can be mapped to well-defined transcripts, 5% match to hypothetical proteins/open reading frames of unknown function and 13% represent unknown transcripts (**Figure 2B**). A total of 18% of the significantly over expressed transcripts possibly represent novel genes and/or alternative transcripts [16] uniquely expressed in the IPF transcriptome. Careful analysis of the over expressed genes in IPF revealed known genes that have been shown to be highly expressed in IPF like S100 calcium binding protein 2, chemokine CXC ligand 14, several collagens, tenascin, metalloprotease 7, and fibronectin. These results confirm previously published IPF and normal expression profiles comparisons [17]–[19]. However, our results also indicate that there are other genes over expressed in our SAGE IPF libraries when compared to normal lung with an unknown role in IPF pathogenesis like syndecan 1(SDC1), suppression of tumorigenicity 5 (ST5, regulator of MAPK1/ERK2 kinase) and centaurin delta 2 (CENTD2); all of which have been associated with lung adenocarcinomas. Interestingly, we did not find significantly down regulated genes in IPF compared to normal lung tissue.

Given that the IPF samples used in this study were selected based on clinical variation in disease progression, we applied a more stringent criteria in order to define a clear molecular signature that would distinguish IPF from normal lung parenchyma. We applied the above mentioned criteria and then selected for the expression of at least 5 counts in more than 75% of all the IPF SAGE libraries. This yielded a list of 293 transcripts tags of which 244 matched to well defined genes (**Table S2**). A T-test analysis showed that the mean expression level of the 293 transcripts in IPF differs significantly from the mean level in normal lung parenchyma (P≤2.7E-31). Furthermore, cluster analysis, including five normal lung SAGE libraries (**Table S1**), indicated that this signature is sufficient to separate IPF from normal lung by a single-linkage hierarchical algorithm (**Figure 2D**), a clear improvement in separation when compared with **Figure 2A**. Interestingly, even when using all 22 SAGE libraries the 293-signature results in a good separation of most IPF from the normal lung parenchyma samples demonstrating the strength of the 293-signature (**Figure S2B**).

### Differentially expressed genes characterizing the *progressive* and *relatively stable* disease groups in IPF

**Figure 3:**
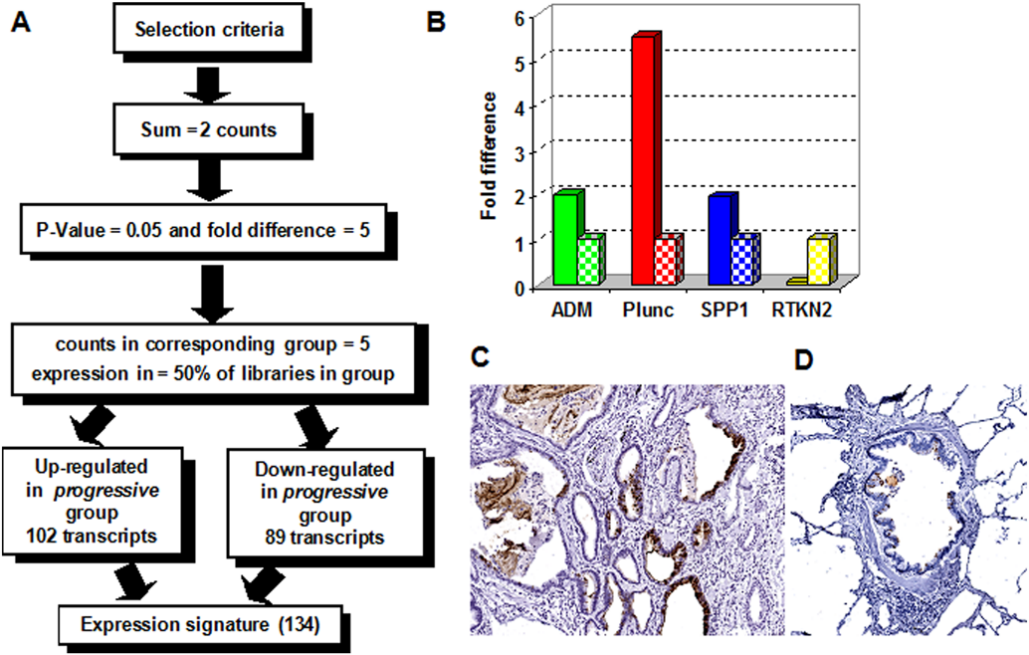
Differentially expressed genes in the lung parenchyma from the relatively stable and progressive IPF. (A) Selection criteria applied in order to find significantly differentially expressed genes. (B) Relative mRNA expression of selected genes. Real-time PCR reactions were performed in triplicate, and the threshold cycle numbers were averaged. Gene expression levels were normalized to GAPDH, and PGK1. The genes ADM (adrenomedullin), Plunc (palate, lung and nasal epithelium carcinoma associated), and SPP1 (osteopontin) were selected as up regulated; and RTKN2 (rhotekin 2) as down regulated in the progressive group. The values obtained for the relatively stable group was arbitrarily set to one to calculate a fold difference. The fold difference in the progressive group is indicated by solid bars and the levels in the relatively stable group are represented by the patterned bars. The differences were not significant as calculated by a Mann-Whitney test. (C) Paraffin-embedded tissue was stained with Plunc antibodies and counterstained with hematoxylin. A representative IPF sample shows strong staining of the secretory/goblet type of bronchial columnar cells (10X magnification). (D) Control bronchial normal lung tissue showed no staining (10X magnification).

After establishing that our newly generated SAGE IPF transcriptome contained sufficient information to distinguish IPF from normal lung parenchyma and other lung diseases, we analyzed the differential expression between progressive and relatively stable IPF. The progressive and relatively stable groups had 446,158 and 508,774 total SAGE tag counts respectively. After applying a filter for a total sum of at least 2 or more tag counts in both groups only 16,089 unique transcript tags were left of which 13,745 were in common between the two groups; 1,268 were only present in the progressive group and 1,076 were only present in the relatively stable group. To identify significant differentially expressed transcript tags that distinguished the two groups, we also selected for a fold difference ≥5, a minimal tag count in the corresponding group ≥5 counts, and a P-value≤0.05 resulting in 243 differentially expressed transcripts. As a final filter, we selected for an expression level of 4 or more counts in at least 50% of the SAGE libraries representing either of the two groups. In this way, we identified 102 transcripts up regulated and 89 down regulated transcripts in the progressive group (**Figure 3A**, **Table 2** **and** **S3**). The up regulated genes in the progressive group includes surfactant protein A1 (SFTPA1) and also members of the MAPK-EGR1-HSP70 pathway that regulates cigarette-smoke induced inflammation [20]. Other up regulated genes are ADM (adrenomedullin), CCL2 (chemokine ligand 2), PTPRF (protein tyrosine phosphatase receptor F) and SPP1 (osteopontin). Interestingly, we found 26 genes among the list of 102 up regulated transcripts that are also associated with various cancers like Heat shock 70KDa protein 1A (HSPA1A), Macropain (PSMA7), Ras homolog gene family member B (RHOB), FK506 binding protein 2 (FKBP2) and Plunc (palate, lung and nasal epithelium carcinoma associated). None of the above mentioned genes, with the exception of SFTPA1, have been previously correlated with disease progression in IPF. Other candidate molecular biomarkers, not necessarily previously implicated in IPF pathogenesis, were selected by IPA analysis among the differentially expressed genes in the progressive group and are listed in **Table 3**. Real-time PCR confirmed the over expression of ADM, Plunc, SPP1, and the down regulation of RTKN2 (rhotekin 2) in a subset of samples (n = 4) used for SAGE library construction representing the progressive group. The values obtained for the relatively stable group (n = 4) was arbitrarily set to one in order to calculate a fold difference (**Figure 3B**). Both ADM and SPP1 have been previously shown to be up regulated in IPF confirming our results [13], [21], [22]. To examine the cellular distribution of Plunc, we analyzed IPF and normal lung tissue by immunohistochemistry. Plunc was found to be mainly expressed in the secretory/goblet type of bronchial columnar cells. In regions of honeycombing there are bronchial/bronchiolar epithelia (including the secretory type) that are strongly staining. It appears that Plunc is also secreted into the mucus that is filling these cystic spaces (**Figure 3C**). No Plunc expression was detected in normal lung tissue (**Figure 3D**).

**Table 2:** Top 50 differentially expressed genes in progressive group

ID	Tag Sequence	Log2 ratio	Symbol	Gene description
1	AGAGGGTGGG	3.5	DNAJB1	DnaJ (Hsp40) homolog, subfamily B, member 1
2	CTGAATGTAC	3.5	MEST	Mesoderm specific transcript homolog (mouse)
3	ATGGTGGGGG	3.3	ZFP36	Zinc finger protein 36, C3H type, homolog (mouse)
4	GTACTAGTGT	3.3	CCL2	Chemokine (C-C motif) ligand 2
5	TCAAGCCATC	3.3	EGR1	Early growth response 1
6	ATTGATGTGT	3.2	SFTPA1	Surfactant, pulmonary-associated protein A1
7	TGCCTCACCT	3.2	PLUNC	Palate, lung and nasal epithelium carcinoma associated
8	AAGAGCGCCG	3.2	HSPA1A	Heat shock 70kDa protein 1A
9	ATGATGTGTA	3.2	SPATS2	Spermatogenesis associated, serine-rich 2
10	AATGTAATCA	3.2	SRI	Sorcin
11	ACATCATACT	3.2	IPO4	Importin 4
12	CCCTTCTATT	3.2	CMAS	Cytidine monophosphate N-acetylneuraminic acid synthetase
13	GAAAAGGGTT	3.2	LAPTM4B	Lysosomal associated protein transmembrane 4 beta
14	TTTAAATAGC	3.2	KLF4	Kruppel-like factor 4 (gut)
15	GCCGATCCTC	3.2	TBCA	Tubulin-specific chaperone a
16	AACTCCCAGT	3.0	GADD45B	Growth arrest and DNA-damage-inducible, beta
17	TGCCAGGTCT	3.0	SFTPA2	Surfactant, pulmonary-associated protein A2B, shorter alternative transcript
18	GCCCCGAGCC	3.0	REEP5	Receptor accessory protein 5
19	GTCCGAGTGC	3.0	TM4SF1	Transmembrane 4 L six family member 1
20	ACCACTTATC	3.0	CTSB	Cathepsin B
21	AGCTTCCAGC	3.0	METRNL	Meteorin, glial cell differentiation regulator-like
22	CGGCTGCCCA	3.0	SNCG	Synuclein, gamma (breast cancer-specific protein 1)
23	CTCTCTACTT	3.0	ITGB1BP1	Integrin beta 1 binding protein 1
24	GAAGTTTTAC	3.0	GNS	Glucosamine (N-acetyl)-6-sulfatase (Sanfilippo disease IIID)
25	GGGGGAGGGA	3.0	TMEM30A	Transmembrane protein 30A
26	CTTCTAGGGA	−2.6	SIN3B	SIN3 homolog B, transcription regulator (yeast)
27	GACAGTCACT	−2.6	ARHGEF4	Rho guanine nucleotide exchange factor (GEF) 4
28	GCTGTGCTGG	−2.6	RIP	RPA interacting protein
29	GGCTATACAG	−2.6	YLPM1	YLP motif containing 1
30	GTTGTGTTAA	−2.6	TMEM125	Transmembrane protein 125, internally primed
31	TAAGAAAAAA	−2.6	CHRDL1	Chordin-like 1
32	TAGAGCTTGT	−2.6	NEK4	NIMA (never in mitosis gene a)-related kinase 4
33	TCTGTTACAC	−2.6	PITPNM2	Phosphatidylinositol transfer protein, membrane-associated 2
34	TGCAAGAGAG	−2.6	ARHGAP30	Rho GTPase activating protein 30
35	TGCCATTAAG	−2.6		mitochondrial
36	TGCGTCACCG	−2.6	SMARCA4	SWI/SNF related, matrix associated, actin dependent regulator of chromatin, subfamily a, member 4
37	TGGTCTGGAG	−2.6	MYO18A	Myosin XVIIIA
38	TGTATTTGAA	−2.6	SLC11A2	Solute carrier family 11 (proton-coupled divalent metal ion transporters), 2
39	TGTTAACAGA	−2.6	NHLRC2	NHL repeat containing 2
40	TTTTGAAGAA	−2.6	GTF2I	General transcription factor II, i
41	ACCTCCCCAC	−2.3	CYP2B7P1	Cytochrome P450, subfamily B, polypeptide 7 pseudogene 1
42	TTTACTTTGG	−2.3	C9orf61	Chromosome 9 open reading frame 61
43	TTTGAATCAG	−2.3	FAM46A	Family with sequence similarity 46, member A
44	ACGCTCTCGA	−2.3	CD37	CD37 antigen
45	AGCCACCTCA	−2.3	ZCCHC4	Zinc finger, CCHC domain containing 4
46	GCCCAGGGAA	−2.3	ARRDC2	Arrestin domain containing 2
47	TACTCAGAGG	−2.3	PAK2	P21 (CDKN1A)-activated kinase 2
48	TATATTTCCA	−2.3	P29	CCNDBP1 interactor
49	TCCAAACCCC	−2.3	DST	Dystonin
50	TGGTGATGAT	−2.3	TAL1	T-cell acute lymphocytic leukemia 1

The top 25 up and down regulated genes in the progressive disease group are indicated. A complete list of the differentially expressed 191 transcript tags and their corresponding matching genes group is presented in **Table S3**.

**Table 3:** Candidate Biomarkers for disease progression based on IPA database survey

Gene Symbol	Gene Description	Location	Fold change	B	BAL	P/S	Sp	L
HLA-DQA1	major histocompatibility complex, class II, DQ A1	Plasma Membrane	14	x				x
MEST	mesoderm specific transcript homolog (mouse)	Extracellular Space	11		x			x
CCL2	chemokine (C-C motif) ligand 2	Extracellular Space	10	x	x	x		x
ZFP36	zinc finger protein 36, C3H type, homolog	Nucleus	10	x				x
HSPA1A	heat shock 70kDa protein 1A	Cytoplasm	9	x	x			x
KLF4	Kruppel-like factor 4 (gut)	Nucleus	9	x				x
PLUNC	palate, lung and nasal epithelium carcinoma ass.	Extracellular Space	9		x		x	x
SRI	Sorcin	Cytoplasm	9		x			x
ADM	adrenomedullin	Extracellular Space	8	x		x		x
AQP1	aquaporin 1 (Colton blood group)	Plasma Membrane	8	x				x
CTSB	cathepsin B	Cytoplasm	8	x	x	x		x
DYNLT1	dynein, light chain, Tctex-type 1	Cytoplasm	8		x			x
GADD45B	Growth arrest and DNA-damage-inducible, beta	Cytoplasm	8	x				x
TMBIM1	transmembrane BAX inhibitor motif containing 1	Unknown	8		x			x
PSMA7	proteasome (macropain) subunit, alpha type, 7	Cytoplasm	7	x	x	x		x
UBB	ubiquitin B	Cytoplasm	7	x				x
CD14	CD14 molecule	Plasma Membrane	6	x	x	x		x
CDC42	cell division cycle 42	Cytoplasm	6	x	x			x
LGALS3	lectin, galactoside-binding, soluble, 3	Extracellular Space	6	x	x	x		x
NAGLU	N-acetylglucosaminidase, a-Sanfilippo IIIB	Cytoplasm	6	x	x	x		x
PTPRF	Protein tyrosine phosphatase, receptor type, F	Plasma Membrane	6	x	x	x		x
SPP1	secreted phosphoprotein 1 (osteopontin)	Extracellular Space	6	x		x		x
APOA1BP	apolipoprotein A-I binding protein	Extracellular Space	5		x			x
CD74	CD74, major histocompatibility complex II	Plasma Membrane	5	x				x
CD276	CD276 molecule	Plasma Membrane	5	x				x
EIF3A	eukaryotic translation initiation factor 3A	Cytoplasm	5	x				x
FKBP2	FK506 binding protein 2, 13kDa	Cytoplasm	5		x			x
FOXA1	forkhead box A1	Nucleus	5	x		x		x
PDIA4	Protein disulfide isomerase family A, member 4	Cytoplasm	5	x	x	x		x
RHOB	ras homolog gene family, member B	Cytoplasm	5	x				x
TBCA	Tubulin folding cofactor A	Cytoplasm	5		x			x
TES	testis derived transcript (3 LIM domains)	Plasma Membrane	5		x			x
VDAC3	Voltage-dependent anion channel 3	Cytoplasm	5	x		x		x
ACTR3	ARP3 actin-related protein 3 homolog (yeast)	Plasma Membrane	4	x	x			x
DST	dystonin	Plasma Membrane	−5	x	x	x		x
TAL1	T-cell acute lymphocytic leukemia 1	Nucleus	−5	x				x
AHNAK	AHNAK nucleoprotein	Nucleus	−6	x		x		x
BIRC6	baculoviral IAP repeat-containing 6 (apollon)	Cytoplasm	−6	x		x		x
EMR4	Egf-like, mucin-like, hormone receptor 4	Plasma Membrane	−6	x				x
ITPKB	inositol 1,4,5-trisphosphate 3-kinase B	Cytoplasm	−6	x				x
MGAT4A	mannosyl-glycoprotein, transferase 4A	Unknown	−6	x		x		x
SIN3B	SIN3 homolog B, transcription regulator (yeast)	Nucleus	−6	x		x		x
SMARCA4	SWI/SNF related, matrix associated, actin dependent regulator of chromatin A4	Nucleus	−6	x				x
YLPM1	YLP motif containing 1	Nucleus	−6	x		x		x
ARHGEF10	Rho guanine nucleotide exchange factor 10	Cytoplasm	−7		x			x
SAFB2	scaffold attachment factor B2	Unknown	−7	x		x		x
KIAA1217	KIAA1217	Cytoplasm	−9	x		x		x

Based on the literature available in the IPA database the cellular localization and detection in bodily fluids/tissue is indicated. Fold change is represented as the difference in expression level between the progressive and relatively stable group. B = blood; BAL = Bronchoalveolar Lavage Fluid; P/S = Plasma/Serum; SP = Sputum; L = Lung.

### A molecular signature for disease progression in IPF

**Figure 4:**
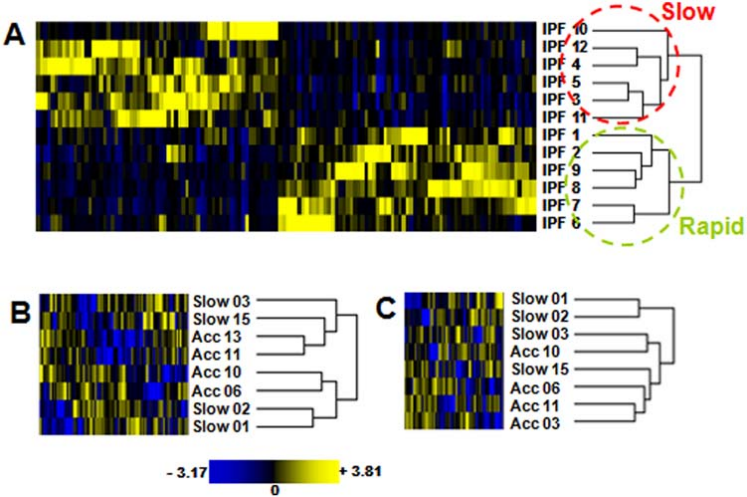
Heat map SAGE molecular signature. (A) Unsupervised clustering of gene expression patterns of IPF lung SAGE libraries described in Table S1, based on the expression signature of 134 transcripts showing a clear distinction between relatively stable (slow) and progressive (rapid) IPF. (B–C) Hierarchical clustering of 8 IPF samples previously identified as a slow and accelerated variants [13] based on 90 (B) or 58 (C) genes in common with the SAGE 134 molecular signature.

To determine if the identified 191 differentially expressed genes associated with rapid progression in IPF (102 up and 89 down regulated) represent a molecular signature, we selected for genes with a P-value<0.05, and analyzed the significance of the difference in mean expression level in both groups and determined that the expression level of 134 of the 191 genes was sufficient to correctly distinguish the progressive from the relatively stable group (Student T-test P-values between 6.5E-3 and 2.4E-5). This expression signature was tested by an unsupervised hierarchical clustering of all IPF lung SAGE libraries used in this study showing a clear distinction between the progressive and relatively stable groups (**Figure 4A**). Interestingly, a study by Selman and colleagues described an accelerated and slowly progressive variant of IPF [13]. The sample size in the Selman microarray based study is small but offers an opportunity to test our 134-signature in an independent cohort. We found 90 genes (67%) in common with our 134 expression signature that were represented on the custom Affymetrix oligonucleotide microarrays [13]. Cluster analysis using those 90 genes was insufficient to clearly distinguish the accelerated variant from the slow variant (**Figure 4B**). Analysis of the significance of the difference in mean expression level between the accelerated and slow variant group among the 90 genes tested, demonstrated that only 58 out of the 90 genes were significant (Student T-test P-value of 8.0E-3). This prompted us to repeat the hierarchical clustering using a smaller set of genes and the results show an improved separation of both groups (**Figure 4C**). It is possible that when using the full progressive IPF signature of 134, the clustering will be even more definitive for the accelerated and slow variant. The clustering ‘behavior’ of the dataset might simply reflect the differences in definition of the accelerated and slow variant between Selman's study and our study. The main distinction being that the slow variant group in Selman's study included subjects with more than 24 months of symptoms while in our study we selected subjects based on their PFT values within a 12 months period following the initial biopsy. Though the preliminary results are promising, the small sample size does not support any formal conclusions regarding the classification strength of the proposed 134-expression signature.

### Pathway analysis of the IPF transcriptome and biomarker selection

Ingenuity pathway analysis (IPA) was applied to select for the main canonical pathways represented in the 1,121 transcript list of over expressed genes in the IPF SAGE Transcriptome, using a Fisher's exact test with a P-value threshold of 0.05. These pathways are the IGF-1 signaling, the ERK/MAPK signaling, the protein ubiquitination, the PI13/AKT signaling, the cardiac β-adrenergic signaling, the actin-cytoskeleton signaling, the integrin signaling, and the NRF2-mediated oxidative stress response pathway (**Figure 2C**). Some of the canonical pathways identified in this study have been previously implicated in IPF [23], [24]. Biomarker analysis using the IPA software identified 33 candidate biomarkers in the 293-IPF SAGE transcript signature. Expression of these genes has been detected in various bodily fluids like blood, bronchoalveolar lavage fluid, plasma/serum, sputum, and lung tissue in various diseases (**Table S4**). Two genes located in the extracellular space have been detected in sputum as well as in other bodily fluids; complement factor H (CFH) and metallopeptidase inhibitor 1 (TIMP1). CFH is secreted into the bloodstream and has an essential role in the regulation of complement activation, and it acts as an adrenomedullin binding protein [25]. The metallopeptidase inhibitor 1 has been previously detected in interstitial macrophages in human IPF samples [26] and is a key player in the fibrogenic response to bleomycin in C57BL/6 mice [27], [28]. The proteins encoded by the TIMP gene family are natural inhibitors of the matrix metalloproteinases (MMPs), a group of peptidases involved in degradation of the extracellular matrix. Though it is unmistakable that MMPs play an important role in IPF pathogenesis the exact mechanism how TIMP1 is activated is still unresolved [29]. Recently it has been shown that the over expression of TIMP1 was an independent prognostic marker in patients with non-small cell lung carcinoma [30].

### Gene Ontology, Pathway and Network analysis of significant differentially expressed genes among patients with progressive IPF

**Figure 5:**
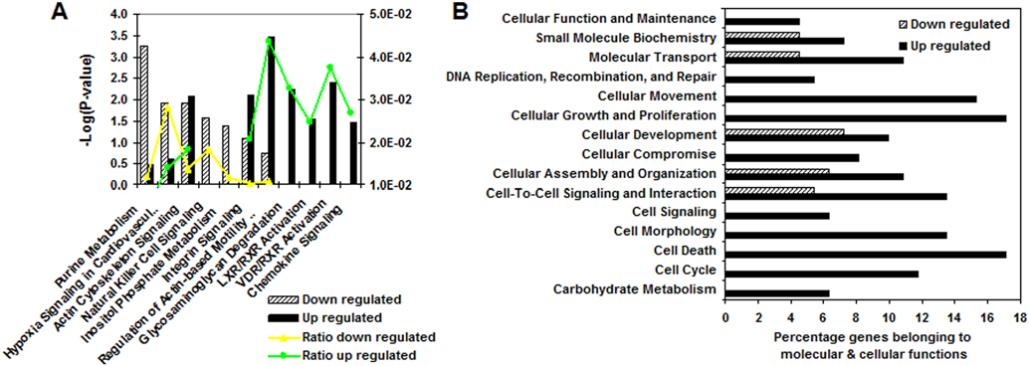
Biological differences between progressive and relatively stable disease groups in IPF. (A) Ingenuity Canonical Pathway analysis showing the most significant pathways associated with the datasets of up and down regulated genes in the progressive group. The significance of the association between the dataset and the canonical pathway was measured as a ratio (number of genes from the dataset that map to the pathway divided by the total number of molecules that exist in the canonical pathway). A Fischer's exact test was used to calculate a P-value. (B) Main molecular and cellular functions significantly associated with the datasets of up and down regulated genes in the progressive group according to the IPA functional analysis tool.

The identified differentially expressed genes in the progressive group offer insight to the possible pathways and cellular processes that might be involved in IPF progression. For a systematic and unbiased analysis we used the Ingenuity Pathway Analysis program to explore the list of differentially expressed genes. **Figure 5A** depicts the top canonical pathways that are significantly associated with our dataset and are highly represented in either the up regulated or down regulated list of genes. The significance is determined by a high ratio (or percentage of genes in pathway found in the gene list) and by a high negative logarithm of the P-value; indicating that the pathway is significantly associated with the data and that a large portion of the corresponding canonical pathway may be affected.

The most prominent pathways in the progressive group (up regulated list of genes) are integrin signaling, regulation of actin-based mobility (cell migration), glycosaminoglycan (mucopolysaccharides) degradation, (LXR/VDR) retinoic X receptor (RXR) activation and chemokine signaling; suggesting an important role for integrin signaling, immune function, bone metabolism and vitamin D3/RXR activation in disease progression. The glycosaminoglycan degradation pathway is a process that seems to be significantly associated with increased disease progression (**Figure 5**). Glycosaminoglycans (mucopolysaccharides) are carbohydrate molecules or complexes of protein and carbohydrate that form the ground substance of connective tissue. One of these carbohydrates is hyaluronic acid. All substances passing to and from cells must pass through the ground substance. Variations in its composition and viscosity may therefore have an important influence on the exchange of materials between tissue cells and the blood. Another finding is the association of the nuclear RXR (retinoic X receptor)/VDR (vitamin D receptor) activation pathway with disease progression. The nuclear RXR can regulate transcription by forming complexes with other nuclear factors and can be activated by retinoid acid. This pathway has until now been unexplored in pulmonary fibrosis, however it is important to note that VDR-deficient mice failed to develop experimental allergic asthma, suggesting an important role for the vitamin D endocrine system in the generation of Th2-driven inflammation in the lung [31].

**Figure 6:**
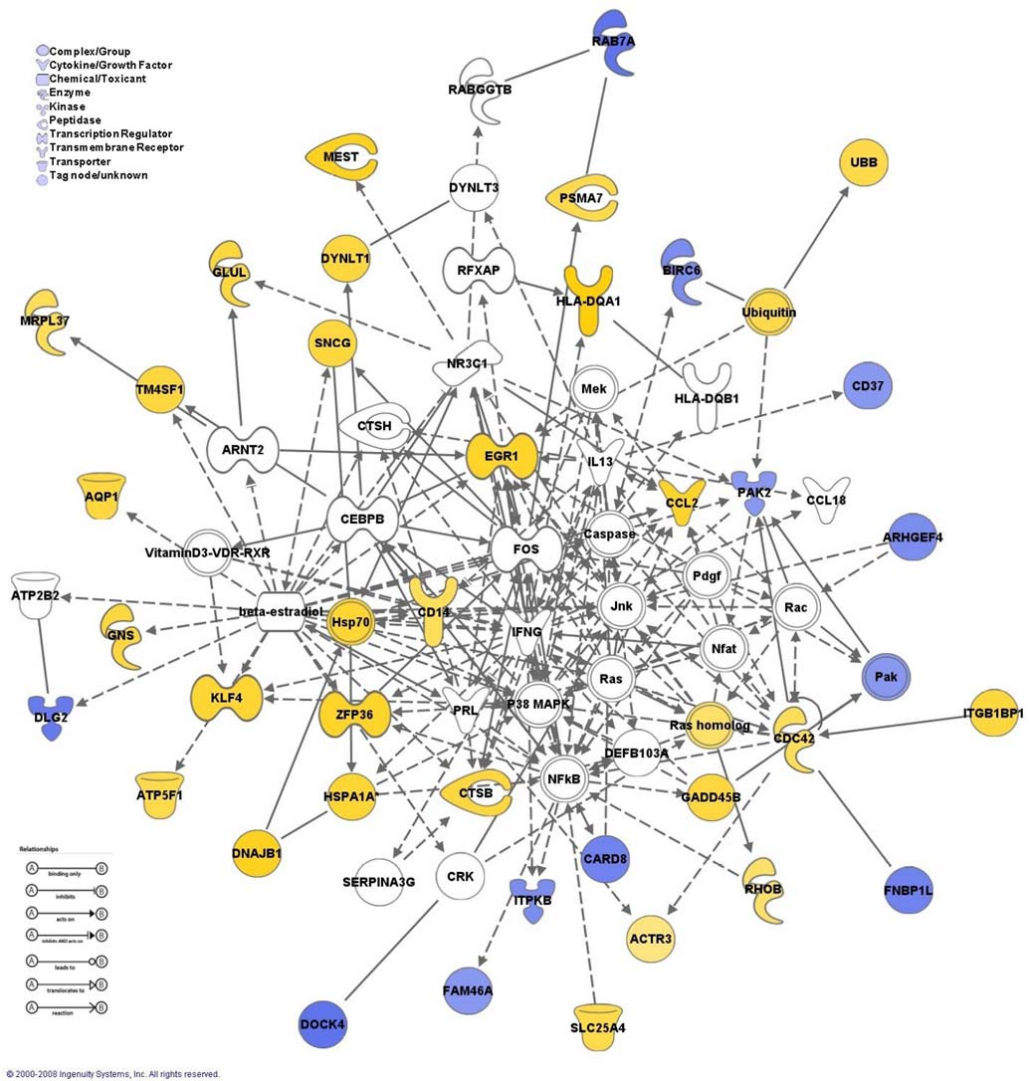
Network analysis. The network map represents the interaction between members of two networks highlighting the crosstalk between the multiple differentially expressed genes in the progressive group. Nodes represent genes, and theirs shapes represent the functional classes of the gene products. Solid lines indicate a direct interaction and dashed lines indicate an indirect interaction.

Another clear distinction can be seen between the two groups of patients with IPF when analyzing the percentage of genes associated with various molecular and cellular functions (**Figure 5B**). During disease progression, an increase is detected in genes associated with cellular growth and proliferation, cellular compromise (stress), cell signaling, cell morphology, cell death, cell cycle and cell movement; molecular functions usually associated with cancer. These results were confirmed by performing a Network analysis. This analysis identified five partially overlapping networks (**Table 4**) in our list of differentially expressed genes, highlighting similar molecular functions associated with the corresponding networks. The overlap between Network 1 and 3 is depicted in **Figure 6** in which the central role for genes like p38 MAPK, NFκB, HSP70, EGR1, CCL2 and Ras homolog can be easily detected.

**Table 4:** Functional network analysis of differentially expressed genes in the progressive group

ID	Molecules in Network	Score (1)	Number Focus Genes	Top Functions	Number associated genes (2)	P-Values (3)
1	ARHGEF4, BIRC6, CARD8, Caspase, **CCL2**, **CD14**, **CDC42**, **DNAJB1**, **EGR1**, FAM46A, FNBP1L, **GADD45B**, Hsp70, **HSPA1A**, **ITGB1BP1**, ITPKB, Jnk, **KLF4**, Mek, Nfat, NFkB, P38 MAPK, Pak, PAK2, Pdgf, Rac, Ras, Ras homolog, **RHOB**, **SLC25A4**, **SNCG**, **UBB**, Ubiquitin, VitaminD3-VDR-RXR, **ZFP36**	43	21	Cell Signaling Cell Death Cancer	10 14 14	1.45-E05 – 1.2E-02 3.67E-05 – 1.59E-01 1.04E-04 – 2.02E-02
2	**ADM**, Akt, ANXA7, Ap1, ARNT, **ATP6V0E1**, ATPase, BAT1, Ck2, **CLEC2B**, **COL4A2**, F Actin, HIF3A, Histone h3, Insulin, KIF1C, LDL, **LGALS3**, Mapk, MTUS1, MYH9, MYO9B, **NFIL3**, **PBEF1**, PDGF BB, PI3K, Pkc(s), **PTPRF**, SH3BP2, **SLC2A3**, **SPP1**, TAL1, Tgf beta, TLN1, Tni	33	17	Hepatic System Disease Gene ExpressionCardiovascular System Development and Function	3 7 9	1.18E-05 – 8.49E-02 8.2E-05 – 3.59E-02 1.48E-04 – 7.0E-02
3	**ACTR3**, **AQP1**, ARNT2, ATP2B2, **ATP5F1**, beta-estradiol, CCL18, CD37, CEBPB, CRK, **CTSB**, CTSH, DEFB103A, DLG2, DOCK4, **DYNLT1**, DYNLT3, FOS, **GLUL**, **GNS**, **HLA-DQA1**, HLA-DQB1, IFNG, IL13, **MEST**, **MRPL37**, NR3C1, PRL, **PSMA7**, RAB7A, RABGGTB, RFXAP, SERPINA3G, **TM4SF1**	30	16	Gene ExpressionCancerTumor Morphology	11166	6.38E-09 – 1.33E-032.68E-08 – 1.41E-032.68E-08 – 6.24E-04
4	ABCB1B, ACSS1, ALDH1A7, ANXA7, **AQP1**, BDKRB1, Ca2+, DMN, DST, DTNA, ELF3, F2, HPSE, KLF6, KLF15, M6PR, **NAGLU**, **PLUNC**, POLR3H, **RARRES1**, RCP9, retinoic acid, **RPL6**, Ryr, SCMH1, SERPINB2, **SLC2A3**, **SNCG**, SOD3, SP1, **SRI**, TGM1, **TM4SF1**, TNF, VEGFA	25	14	CancerCellular MovementCell-to-Cell Signaling and Interaction	211411	1.28E-07 – 1.64E-011.28E-07 – 1.25E-031.45E-07 – 1.35E-03
5	amino acids, AREG, BCL2L1, **CMAS**, COG1, COG2, COG3, COG4, COG5, COG6, COG7, COG8, DUSP4, DUSP6, ELF3, ERBB2, ERRFI1, HGF, HIPK2, IL11, ILK, IRF8, Mek1/2, Mlcp, NEK4, PDE8A, PPP1R12A, **PPP1R12B**, PRKG1, PTPRE, ROCK1, ROCK2, **SERP1**, **VDAC3**, **WBP5**	14	9	Amino Acid MetabolismPost-Translational ModificationSmall Molecule Biochemistry	121415	3.27E-10 – 1.54E-033.27E-10 – 2.01E-033.27E-10 – 4.02E-03

Up regulated genes are indicated in **bold**. Down regulated genes are underlined. Genes not altered in our signature are indicated in plain text. (1) Negative logarithm of the P-value; indicating the likelihood that the focus genes within a network are grouped as a result of random chance; using a confidence level of 99% IPA regards a score ≥2 as significant. (2) Number of significantly associated genes with the corresponding molecular function. (3) Range of significances of the associated genes for the corresponding molecular function.

## Discussion

Our results indicate that molecular signatures of gene expression appear to be useful in the identification of the presence and predictive of the activity of IPF. We have shown that molecular signatures can distinguish IPF from both normal lung and other chronic lung diseases. Moreover, our findings suggest that molecular signatures from lung parenchyma at the time of diagnosis appear to be helpful in predicting disease progression and may prove valuable in predicting the activity of IPF.

Genome-wide analyses of gene expression have facilitated the identification of gene expression patterns or signatures revealing the complexity of human cancer. Most of the work using large scale gene expression data has been focused on discovering gene expression profiles that can lead to a better understanding of tumor development and proliferation. The strength of gene expression analysis has been shown by the ability to identify new cancer subtypes and predict clinical outcome [32]. A prognostic gene expression signature has been proposed for survival in early-stage lung cancer [33] and was recently validated in a large, training-testing, multi-site, blinded study [34]. Gene expression profiling has also allowed the prediction of breast cancer recurrence [35] which has ultimately lead to the development of the Mammaprint, a clinical test based on a 70-genes signature that predicts the risk of metastasis in breast cancer patients [36].

While gene expression profiling has proven to be a powerful tool for the identification of specific gene patterns and pathways associated with certain types of human cancers, our findings suggest that these molecular signatures may also prove useful in understanding complex lung diseases, like IPF. The increase of the protein ubiquitination pathway could be associated with an increase of apoptosis of epithelial cells but has not been extensively studied in IPF. There are few studies implicating the PI3/AKT signaling pathway in IPF. Bleomycin-induced pulmonary fibrosis studies in mice have shown activation not only of TGF-beta but also phosphatidylinositol 3-kinase (PI3K) and protein kinase B via a Semaphorin (SEMA) 7A-dependent mechanisms, and PKB/AKT inhibition diminished TGF-beta-induced fibrosis [37]. SEMA 7A was not found to be differentially expressed in our dataset though many family members and its receptor intergrin beta are involved in the transcriptional profile of IPF. It has been shown that collagen accumulation can be reduced by the administration of PI3K inhibitors [38], implying that the PI3K/AKT pathway might play an important role in pulmonary fibrosis. Deregulation of the PI3K/PTEN/AKT pathway is one of the most common altered pathways in human malignancy. Significant advances have been made in the understanding of the AKT signaling pathway in oncogenesis and in the development of small molecule inhibitors. Whether this pathway could be targeted in human pulmonary fibrosis remains to be established and could offer new treatment opportunities. The integrin signaling pathway is anticipated to be associated with pulmonary fibrosis since integrins are the primary extracellular matrix (ECM) receptors mediating ECM remodeling [39]. In response to changes in the ECM, integrin signaling also regulates many other interrelated cellular processes like proliferation, survival, cell migration and invasion. However, further studies in larger cohorts, using either, real-time PCR, a customized SAGE signature array or tissue-array, are needed to validate the importance and relevance of these findings for early diagnosis and disease management.

Our results may have a significant impact in the development of early biomarkers for IPF. Identifying biomarkers that could reduce the time to diagnosis may create a window of opportunity for therapeutic intervention, especially in a disease like IPF where the diagnosis is often delayed. While our transcriptional signature for disease progression was developed using lung biopsy samples, 47 of the 134 gene products that were associated with clinical progression have been detected in body fluids in various diseases (such as blood, plasma/serum, bronchoalveolar lavage fluid or sputum) according to Ingenuity Pathway Analysis software. Although for many of these 47 genes the biological function and role in IPF pathogenesis is unknown, these genes and gene products could potentially serve as biomarkers for this disease. Genes like ADM (adrenomedullin), CCL2 (chemokine ligand 2), PTPRF (protein tyrosine phosphatase receptor F) and SPP1 (osteopontin) play a role in the migration of smooth muscle cells and cell proliferation and/or invasion implying a potentially more important role of these processes in disease progression. The chemokine CCL2 have been previously detected in metaplastic epithelial cells and vascular endothelial cells of IPF cases and it was proposed that CCL2 may play a key role in the irreversible progression of IPF [40]. In addition, a decrease of lung fibrosis was detected in CCL2 null mice when exposed to bleomycin [41], [42]. What's more, CCL2 has been shown to be elevated in human bronchoalveolar lavage fluid from patients with IPF [42], [43]. The protein was measured in plasma as well and it was shown that there was no significant difference between IPF patients and normal controls [44]. Our results indicate that CCL2 is a potential marker of disease progression in IPF. Whether the plasma levels of CCL2 correlates with disease progression remains unknown [44]. Interestingly, SPP1 have been localized to the alveolar epithelial cells in IPF lungs, was also significantly elevated in bronchoalveolar lavage fluid from IPF patients [21] and, has been detected in plasma from patients with idiopathic interstitial pneumonia [45]. Previous studies have shown that SPP1 null mice clearly develop less fibrosis when exposed to bleomycin. It was suggested that SPP1 is secreted by the epithelial cells and has a profibrotic effect [21].

Some of these potential biomarkers genes have been implicated in human cancers. Heat shock 70KDa protein 1A (HSPA1A) is up regulated in brain, lung, and liver cancer. Macropain (PSMA7) is increased in brain, breast, and stomach cancer, and plays an important role in colorectal cancer progression providing a unique target for drug development. The Ras homolog gene family member B (RHOB), a Rho GTPase, is up regulated in brain and breast cancer though down regulated in lung neoplasms. These GTPases are crucial regulators of the actin cytoskeleton and also play an important role in membrane trafficking. Associated with lung cancer are FK506 binding protein 2 (FKBP2) and Plunc (palate, lung and nasal epithelium carcinoma associated). The latter gene belongs to the PLUNC family of proteins postulated to play a role in innate immune response and is uniquely expressed in the upper respiratory tract. Studies in cystic fibrosis have shown a significant elevation of Plunc expression in diseased airways [46], [47]. As Plunc can be detected in sputum [48] and bronchoalveolar lavage fluid, it appears to be an ideal candidate biomarker for disease progression in IPF. SAGE and microarray analysis have recently indicated that Plunc is a novel marker that distinguishes gastric hepatoid adenocarcinoma from primary hepatocellular carcinoma [49].

The extensive SAGE IPF transcriptome presented in this investigation demonstrates the complexity and scope of the biological activity involved in IPF. Some of the pathways identified by SAGE profiling have not been previously associated with IPF. Network and pathway analyses have also shown that various signaling pathways can interact or even partially overlap with each other, thereby suggesting that IPF may be the result of multiple, consecutive (or interactive) biological events, possibly triggered by environmental stimuli. However, despite this biological complexity, our findings clearly illustrate that molecular signatures of gene expression in IPF may prove helpful in predicting disease progression among those with IPF. Molecular and cellular functions like cell proliferation, migration, invasion and cell morphology appear to be over represented in the more progressive IPF group; a striking similarity with human cancers. The association with disease progression and the identifiable heterogeneity seen within samples emphasize the importance and the need for an extensive molecular classification of IPF and other forms of interstitial lung disease. The recognition that IPF may have different subtypes that can be distinguished by their molecular patterns could identify novel therapeutic targets and personalize the clinical approach to this complex group of diseases.

## Materials and Methods

### Study Population

Lung tissue was obtained from patients with IPF who had a definitive diagnosis based on UIP pathology from a surgical lung biopsy [1], [2]. Flash frozen surgical lung biopsy specimens, were obtained from 12 patients with IPF who were undergoing initial diagnostic evaluation and were not being treated for their IPF. The protocol was approved by the Institutional Review Board from National Jewish Health and written informed consent was obtained where required. Further processing of the frozen samples was performed at the National Institutes of Health (NIH). This research activity was approved by the Office of Human Subjects Research at the NIH. The 12 specimens were specifically obtained from two groups of patients; progressive IPF or relatively stable IPF. In the progressive group (n = 6), the percent predicted forced vital capacity (FVC) and the percent predicted diffusing capacity of carbon monoxide (DLCO) declined significantly up to 12 months following biopsy (respectively ≥10% and ≥15% ). The relatively stable group (n = 6) had a relatively uneventful eventless course over the 12 months following surgical lung biopsy with a decline in percent predicted FVC<10% or a decline in percent predicted DLCO<15%. No patient in either group received treatment for IPF prior to lung biopsy.

### RNA isolation and SAGE library construction

Total RNA was extracted from frozen lung tissue using the RNAgents total RNA isolation system (Promega, Madison, WI, USA). The quality of total RNA was analyzed using the RNA 6000 Nano Labchip kit on a 2100 BioAnalyzer (Agilent Technologies, Santa Clara, California). On average 1 to 5 µg of total RNA as determined by the Ribo-Green RNA Quantification kit (Molecular Probes, Eugene, OR, USA) was used to construct SAGE libraries from 12 IPF samples using *Nla* III as the anchoring enzyme and *BsmF* I as the tagging enzyme according to a micro-SAGE protocol [50]. The SAGE library clones were arrayed and inserts were purified and sequenced at Agencourt Bioscience Corporation. The SAGE 2000 software version 4.5 (available at http://www.sagenet.org) was used to extract SAGE tags from the original sequence files, remove duplicate ditags, remove linker sequences, remove one base pair variations of linker sequences and tabulate the occurrence of each tag. Tag sequences, tag counts and gene associations were stored in a Microsoft Access relational database for subsequent analysis. The complete SAGE IPF dataset have been deposited in the GEO database (GSE11665). Other SAGE profiles used in this study were downloaded from the GEO (http://ncbi/geo/) or the SAGE Genie [51] website and are depicted in **Table S1**. P-values for differentially expressed transcripts were calculated according to the sequence odds ratio and significant test (http://cgap.nci.nih.gov/SAGE). Similar results were obtained when using the SAGE software Monte Carlo approach or the significant test available as part of the DiscoverySpace application [52].

### Hierarchical Clustering

The open source clustering software Cluster 3.0 was used for gene expression data analysis. Cluster 3.0 is an enhanced version of Cluster [53] built for the Microsoft Windows platform. The Cluster program is based on a modified Pearson correlation, and was applied to the normalized SAGE data. Starting with a dataset of 149,291 unique transcript tags, a second filter selecting for a project total (sum expression level all 22 libraries included in this study) of ≥10 counts was applied reducing the amount of transcript tags to 23, 649. This SAGE dataset was then filtered for at least five observations across the 22 libraries, with an absolute value ≥2 and a maximum minus minimum value ≥2. These settings produced a data set of 11,467 transcript tags. This data set was subsequently adjusted by performing median centering and normalization. This procedure resulted in a median-polished (i.e. all row and column-wise median values are close to zero) and normal (i.e. all row and column magnitudes are close to 1.0) dataset. Next, the dataset was analyzed by applying a correlation centered complete linkage clustering, which assembles the dataset into a tree. Items joined by short branches are very similar, whereas longer branches represent decreasing similarities. Results were displayed with the TreeView program [53]. For confirmation of the results and subsequent clustering of small datasets or microarray data, the MultiExperiment Viewer (Version 4.1, January 18^th^, 2008) was used [54] as well as a modified version specific for SAGE data analysis [55].

### Real-time PCR

Equal amounts of total RNA (5 µg) were used in a 20 µl cDNA synthesis reaction primed with oligo-dT (Superscript II; Invitrogen, Carlsbad, CA, USA). Control reactions were prepared in parallel without reverse transcriptase. Prior to cDNA synthesis, residual genomic DNA was removed from total RNA with a DNase I treatment (DNA-free; Ambion, Austin, TX, USA). Quantitative PCR was performed with a 7900TH Fast Real-Time PCR system (Applied Biosystems, Foster City, CA, USA) using SYBR-Green. PCR reactions were performed in triplicate, and the threshold cycle numbers were averaged. Gene expression levels were normalized to ACTB (actin, beta), and PGK1 (phosphoglycerate kinase 1). The relative expression levels were calculated in comparison to the levels in total RNA from normal lung (Ambion, Austin, TX) according to the Comparative Ct method in which the relative expression equals 2-ΔΔCt. PCR primers were designed using the Primer 3 interface (http://www.bioinformatics.nl/cgi-bin/primer3plus/primer3plus.cgi).

### Immunohistochemistry

Five micron sections of paraffin-embedded tissue were deparaffinized using 5 minute incubations in Xylene followed by 100% and 95% ethanol. Slides were rinsed in distilled water and then incubated for 30 minutes in a 3% aqueous solution of hydrogen peroxide at room temperature. Slides were rinsed in distilled water and then transferred to a Citrate buffer at pH 6.0 for antigen retrieval in a pressure cooker at 125°C for 5 minutes followed by a gradual cooling back to room temperature. Slides were rinsed in distilled water, and then washed for 5 minutes in Tris-buffered saline with 0.1% Tween (TBS-T). Washed slides were incubated in Serum-free protein blocking buffer (DAKO, Carpenteria, CA) for 30 minutes. Blocking buffer was removed without washing and 100 ul of biotinylated primary antibody (#BAF1897, 1:50 dilution in TBS-T, R&D Systems, Minneapolis, MN) was applied to each slide. Slides were incubated in primary antibody solution overnight at 4°C. Slides were washed for 5 minutes in TBS-T. Biotin-labeled antibody detection was carried out using the Vectastain RTU ARB reagent (Vector Laboratories, Burlingame, CA) following the manufactures instructions. Staining was visualized with a 5 minute room temperature incubation using DAB chromagen/buffer (DAKO, Capenteria, CA). The color reaction was stopped in distilled water and slides were counterstained for 3 minutes in hemotoxylin, dehydrated in graduated ethanol's and cleared using Xylene prior to cover slipping. All slides were scanned using the Aperio ScanScope XT (Aperio, Vista, CA).

### Gene Ontology, Biomarker selection and Functional Network Analysis

Data were analyzed through the use of Ingenuity Pathways Analysis (Ingenuity Systems®, www.ingenuity.com). Ingenuity Pathway Analysis (IPA) is a web-based application that enables the visualization, discovery and analysis of molecular interaction networks within gene expression profiles. All generated gene lists and corresponding expression levels, represented as the log_2_ ratios, were uploaded within the IPA database for further analysis. Both gene symbols and gene bank accession numbers were used with no apparent differences in results. These genes, called focus genes, were overlaid onto a global molecular network developed from information contained in the Ingenuity knowledge base. The IPA knowledge base represents a proprietary ontology of over 600,000 classes of biologic objects spanning genes, proteins, cells and cell components, anatomy, molecular and cellular processes, and small molecules. ***Networks*** of the focus genes were then algorithmically generated based on their connectivity. The ***Functional Analysis*** of a network identified the biological functions and/or diseases that were most significant to the genes in the network. The network genes associated with biological functions and/or diseases in the Ingenuity knowledge base were considered for the analysis. Fischer's exact test was used to calculate a P-value determining the probability that each biological function and/or disease assigned to that network is due to chance alone. ***Canonical Pathways Analysis*** identified the pathways from the Ingenuity Pathways Analysis library of canonical pathways that were most significant to the dataset. The significance of the association between the dataset and the canonical pathway was measured in 2 ways: 1) a ratio of the number of genes from the dataset that map to the pathway divided by the total number of molecules that exist in the canonical pathway is displayed; 2) Fischer's exact test was used to calculate a P-value. ***Biomarker Analysis*** allows the identification of the most relevant molecular biomarker candidates from a dataset based on contextual information such as mechanistic association with a disease or detection in bodily fluids.

## Supporting Information

Figure S1 Lung function test values for individual samples included in this study. Actual DLCO (A) or FVC (B) values are depicted in a scatter dot plot with mean and standard deviation. The progressive group is represented in red (dots) and the relatively stable group in blue (squares).
(0.45 MB TIF)

Figure S2 Unsupervised Hierarchical clustering analysis of all 22 SAGE libraries based on 11,467 transcripts (A) and based on the 293-gene expression signature (B).
(1.02 MB TIF)

Table S1 Summary SAGE libraries included in this study
(0.03 MB PDF)

Table S2 Genes over expressed in IPF when compared to normal lung.
(0.07 MB XLS)

Table S3 Differentially expressed genes in progressive group
(0.06 MB XLS)

Table S4 Candidate IPF biomarkers.
(0.02 MB XLS)
