# Supplementary figures and images for: Molecular Phenotypes Distinguish Patients with Relatively Stable from Progressive Idiopathic Pulmonary Fibrosis (IPF)
Source: PLoS One. 2009 Apr 6;4(4):e5134. doi: 10.1371/journal.pone.0005134 (PMC2661376; doi:10.1371/journal.pone.0005134)

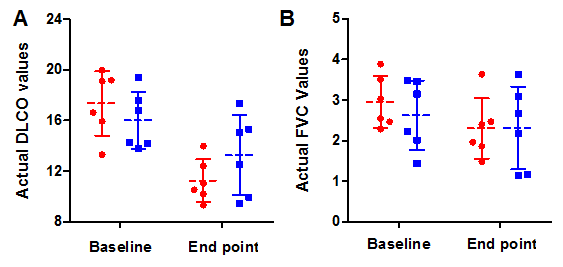

Supplement: Figure S1 — Lung function test values for individual samples included in this study. Actual DLCO (A) or FVC (B) values are depicted in a scatter dot plot with mean and standard deviation. The progressive group is represented in red (dots) and the relatively stable group in blue (squares). (0.45 MB TIF) [file pone.0005134.s001.tif]

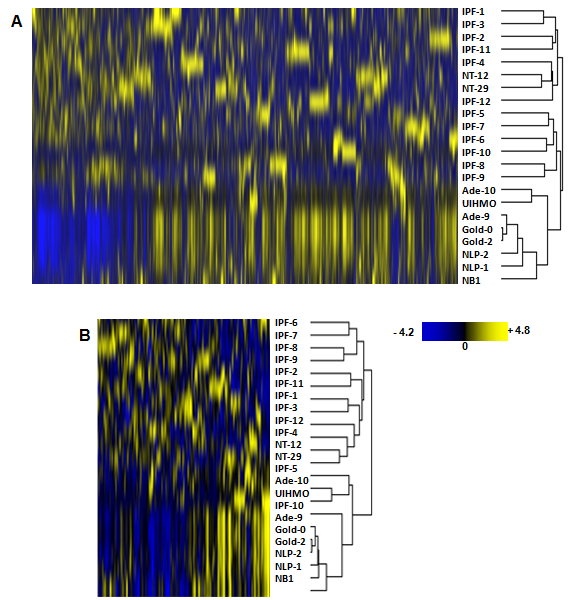

Supplement: Figure S2 — Unsupervised Hierarchical clustering analysis of all 22 SAGE libraries based on 11,467 transcripts (A) and based on the 293-gene expression signature (B). (1.02 MB TIF) [file pone.0005134.s002.tif]
