# Supplementary material for: Molecular Phenotypes Distinguish Patients with Relatively Stable from Progressive Idiopathic Pulmonary Fibrosis (IPF)
Source: PLoS One. 2009 Apr 6;4(4):e5134. doi: 10.1371/journal.pone.0005134 (PMC2661376; doi:10.1371/journal.pone.0005134)
Supplement: Table S1 — Summary SAGE libraries included in this study (0.03 MB PDF) [file pone.0005134.s003.pdf]

Table S1. Summary SAGE libraries included in this study

Sample ID	Age	Gender	Smoking status and other features	Total tags	Unique tags
Ad-9 (1)	-	-	well differentiated adenocarcinoma with lymphoplasmatic infiltration (cancer)	35,916	15,131
Ad-10 (1)	-	-	poorly differentiated adenocarcinoma with lymphoplastic infiltration, microdissected (cancer)	86,887	29,062
UIHMO (1)	-	F	focal fibrosis, chronic inflammation, tumor associated	98,962	32,281
Gold-2 (2)	48 to 76	M/F	samples from 14 smokers, pulmonary function test consistent with moderate COPD	30,070	12,188
Gold-0 (2)	45 to 76	M/F	samples from 12 smokers, pulmonary function test exhibit non-obstruction	29,273	20,193
NB1 (1)	20	M	young adult, normal lung	88,708	24,764
NLP-1 (3)	54 - 84	1 M/3 F	normal lung parenchyma pool of 4	66,214	17,846
NLP-2 (3)	65 - 77	M	normal lung parenchyma pool of 4	64,214	20,003
NT12 (4)	69	M	normal lung parenchyma, non-smoker	55,954	17,850
NT29 (4)	17	M	normal lung parenchyma, non-smoker	79,230	22,409
IPF-1 (4)	67	M	lung biopsy tissue, non-smoker (5)	99,936	32,106
IPF-2 (4)	56	F	lung biopsy tissue, smoker (5)	70,028	22,711
IPF-6 (4)	72	M	lung biopsy tissue , non-smoker (5)	57,446	28,722
IPF-7 (4)	57	M	lung biopsy tissue, smoker (5)	96,954	31,591
IPF-8 (4)	67	M	lung biopsy tissue, non-smoker (5)	86,484	31,186
IPF-9 (4)	70	M	lung biopsy tissue, smoker (5)	97,926	29,450
IPF-3 (4)	64	M	lung biopsy tissue, smoker (6)	113,110	33,071
IPF-4 (4)	67	F	lung biopsy tissue, smoker (6)	107,624	33,987
IPF-5 (4)	64	F	lung biopsy tissue, smoker (6)	90,632	29,887
IPF-10 (4)	67	F	lung biopsy tissue, non-smoker (6)	59,428	26,977
IPF-11 (4)	61	M	lung biopsy tissue, non-smoker (6)	43,432	15,212
IPF-12 (4)	77	M	lung biopsy tissue, smoker (6)	31,932	12,858

(1) SAGE libraries downloaded from the SAGE Genie Website (http://cgap.nci.nih.gov/SAGE).
(2) Gold SAGE libraries described by Ning *et al*., [1]. (3) NLP SAGE libraries described by Lonergan *et al*., [2] and downloaded from the GEO website (http://ncbi/geo/). (4) SAGE libraries generated for this study. (5) And (6) respectively; progressor and relatively stable disease group as defined by clinical characteristics. Smoking status refers to a current or ever smoker.

1. Ning W, Li C-J, Kaminski N, Feghali-Bostwick CA, Alber SM, et al. (2004) Comprehensive gene expression profiles reveal pathways related to the pathogenesis of chronic obstructive pulmonary disease. Proceedings of the National Academy of Sciences 101: 14895-14900.
2. Lonergan KM, Chari R, deLeeuw RJ, Shadeo A, Chi B, et al. (2006) Identification of Novel Lung Genes in Bronchial Epithelium by Serial Analysis of Gene Expression. Am J Respir Cell Mol Biol 35: 651- 661.
